# The efficacy of extracorporeal shock wave for chronic musculoskeletal pain conditions: A protocol of systematic review and meta-analysis of randomized controlled trials

**DOI:** 10.1097/MD.0000000000019705

**Published:** 2020-04-17

**Authors:** Jiawei Qin, Tong Jin, Zexiang He, Lijian Wu, Qiuxiang Lin, Yiheng Lin, Yi Zhang

**Affiliations:** a Department of Rehabilitation Medicine, Quanzhou First Hospital Affiliated to Fujian Medical University, Quanzhou; b Gynecological Minimally Invasive Center, Beijing Obstetrics and Gynecology Hospital, Capital Medical University, Beijing, China.

**Keywords:** effectiveness, ESWT, pain, safety, systematic review

## Abstract

**Background:**

This systematic review is the first one to assess the effectiveness and safety of extracorporeal shock-wave therapy (ESWT) for patients with chronic musculoskeletal pain conditions (CMPC).

**Methods:**

Seven electronic databases were searched for all relevant literature from inception to December 2019, including PubMed, the Web of Science, EMBASE, Cochrane library, China National Knowledge Infrastructure Database (CNKI), Chinese Scientific Journal Database (VIP), and Wanfang database. Only randomized controlled trials (RCTs) of ESWT for chronic musculoskeletal pain will be included. Two reviewers will independently select eligible studies and collected the detailed information, assessed the methodological quality. A third reviewer will join in discussion to solve disagreements. The mean difference (MD) or standard mean difference (SMD) with 95% confidence intervals (CIs) will be presented to demonstrate the effectiveness of ESWT for patients with chronic MSK pain conditions. RevMan 5.4 software will be used for statistical analysis.

**Results:**

This systematic review will explore the effectiveness and safety of ESWT for patients with CMPC. The primary outcome includes pain level, and secondary outcome includes function limitation and adverse events.

**Conclusion:**

It can provide the updated evidence which is of great importance for patients, clinical practice and health related policy maker in ESWT treating CMPC.

## 1 Introduction

Chronic musculoskeletal pain conditions (CMPC) is a common public health problem that affects about 20% adult population worldwide, causing a serious economic burden and high absenteeism rate.^[1,2]^ Over 55% visits to their doctors were for pain-related problem (joint disorders and back problems) in USA.^[3]^ A survey reported that 40% patients did not alleviate or increased pain intensity after visiting emergency department.^[4]^ CMPC was defined as lasting for a duration of more than 3 months and prevalent in a diverse musculoskeletal system impairment including nonspecific neck/back pain, shoulder pain, osteoarthritis (OA), rheumatoid arthritis (RA), and fibromyalgia.^[5]^ CMPC is characterized by the interaction of biophysical, psychological, and social aspects that impair functional activities and participation.^[6]^

Some literatures showed that medications are most commonly used to manage CMPC.^[7]^ However, many patients had no significant pain intensity decrease with medication alone, and medication had a variety of side effects especially for long-term user.^[8]^ Non-pharmacological and noninvasive management are recommended by current related guidelines, including advise to remain active, education, exercise therapy, physical therapy, and cognitive behavior therapy.^[9,10]^ As an alternative physical therapy, extracorporeal shockwave therapy (ESWT) contained an appropriate generator conveying a sequence of single sonic pulses to specific target areas.^[11,12]^ EPSW was widely used in various musculoskeletal disorders and demonstrated to be effective for managing some CMPC such as frozen shoulder, tennis elbow, low back pain, OA knee with few adverse events.^[11–14]^ However, most related researches focus on only 1 pain condition and related systematic reviews were lacked.

This protocol of systematical review and meta-analysis was the first one to evaluate the effectiveness and safety of EPSW in pain relief for patients with CMPC.

## 2 Method

### 2.1 Study registration

This protocol of systematic review has been registered with PROSPERO (CRD42019148814) on https://www.crd.york.ac.uk/PROSPERO/. It has reported according to the Preferred Reporting Items for Systematic Reviews and Meta-Analysis Protocol statement guidelines.

### 2.2 Inclusion criteria

#### 2.2.1 Study types

Only randomized controlled trials (RCTs) of ESWT for chronic musculoskeletal pain will be included in this protocol. Other type studies, such as reviews, letters, case series, case reports, non-RCTs, quasi-RCTs will be excluded.

#### 2.2.2 Patients types

The patients had chronic musculoskeletal pain conditions which last for at least 3 months. Chronic musculoskeletal pain conditions included neck pain, back pain, peripheral joints pain, myofascial pain, rheumatoid arthritis, osteoarthritis, and fibromyalgia.

#### 2.2.3 Intervention types

ESWT should be the only intervention, those studies combined ESWT with other therapies will be excluded. The intervention of control group could be waitlist or any other therapy except the ESWT.

#### 2.2.4 Outcome measurement type

Primary outcome is pain level which could be assessed by many tools such as VAS, NPRS and WOMAC, adverse events will be recorded.

### 2.3 Search strategy

Seven electronic databases were searched for all relevant literature from inception to December 2019, including PubMed, the Web of Science, EMBASE, Cochrane library, China National Knowledge Infrastructure Database (CNKI), Chinese Scientific Journal Database (VIP), and Wanfang database. The detailed search strategy for PubMed is demonstrated in Table 1. The similar strategy will be applied for other electronic databases, and reference of included literatures will also be searched.

**Table 1:**
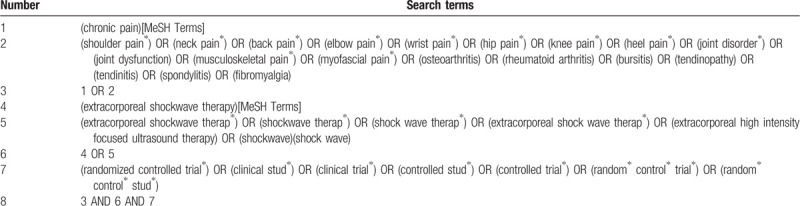
Search strategy applied in PubMed.

### 2.4 Study selection

**Figure 1:**
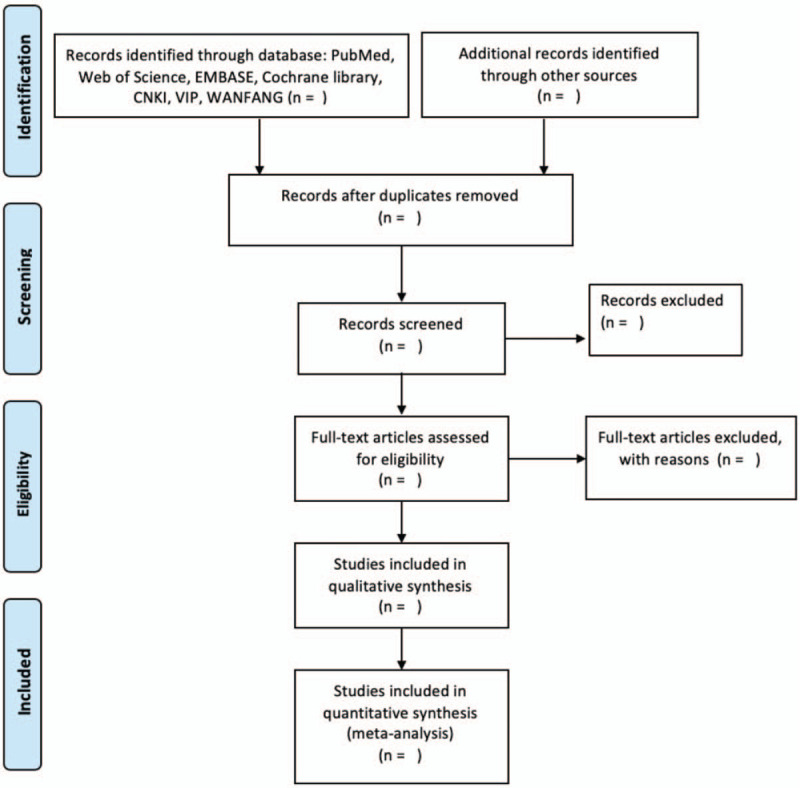
Protocol flow diagram.

Two reviewers will independently extract related information from the included studies by a standard data extraction form. The reviewers check the title information, abstracts and full texts to select eligible studies. A third reviewer will join in discussion to solve disagreements. The protocol flow diagram is shown in Figure 1.

### 2.5 Data extraction and management

Two reviewers independently collected the detailed information of the eligible studies that include authors, published data, research region, sample size, age, symptoms duration, experimental and control group intervention, outcome assessments, adverse events. A third reviewer will join in discussion to solve disagreements.

### 2.6 Risk of bias assessment

Two reviewers assessed the methodological quality of included studies independently by the Physiotherapy Evidence Database (PEDro) scale. The PEDro scale contains 11 items, including random allocation; concealed allocation; baseline comparability; blind subjects; blind therapists; blind assessors; adequate follow-up; intention-to-treat analysis; between-group comparisons; point estimates; and variability. The maximum score of this scale is 10 points which <4 points, 4–5 points, 6–8 points and 9–10 points were categorized as poor quality, fair quality, good quality, and excellent quality. The RCTs rating from fair to excellent quality were suitable for systematic review and meta-analysis of physiotherapy researches. A third reviewer will join in discussion to solve disagreements.

### 2.7 Statistical analysis

#### 2.7.1 Treatment effect measurement

The mean difference (MD) or standard mean difference (SMD) with 95% confidence intervals (CIs) will be presented for continuous outcome data. The risk ratio with 95% CIs will be presented for dichotomous data.

#### 2.7.2 Assessment of heterogeneity

A Chi-Squared and *I*^2^ test were used to assess the statistical heterogeneity among the included studies. The heterogeneity was considered as acceptable if *I*^2^ ≤ 50%. It would be regarded as substantial heterogeneity if *I*^2^ > 50% and *P* value <.1.

#### 2.7.3 Data synthesis

The fixed-effect model will be carried out to pool the outcome data if *I*^2^ ≤ 50%. Otherwise, random-effect model will be used to pool the data, and subgroup analysis will be performed.

#### 2.7.4 Subgroup analysis

Subgroup analysis will be performed when the heterogeneity is substantial. It will be conducted based on different styles of treatment, controls, and outcome assessment tools.

#### 2.7.5 Sensitivity analysis

Sensitivity analysis will be conducted to explore the heterogeneity source and the stability of pooled result by eliminating included studies one by one.

#### 2.7.6 Publication bias

Funnel plot will be employed to assess the potential publication bias if including more than 10 studies. Egger and Begg tests will be performed if funnel plot is asymmetry.

## 3 Discussion

CMPC was defined as ongoing pain occurred in the joints, bones, and tissues of the body which sustained at least 3 months.^[15]^ CMPC was one of the most costly and prevalent disorders globally,^[8]^ it is the major cause for disability and pain that affecting 1 of 5 adults in western society.^[16]^

ESWT are pulsed acoustic waves with a short duration of time but extremely high pressing amplitudes, and low tensile wave components.^[14]^ Some research had documented that ESWT demonstrated positive effects on CMPC. Some reviews have maintained that ESWT had beneficial effects on CMPC, such as chronic pelvic pain, neck pain, tennis elbow, and plantar fasciitis.^[17–20]^ Meanwhile, some experimental data in the effect of ESWT treating chronic pain conditions are somewhat controversial.^[21–23]^ Furthermore, most reviews focused on only 1 disorder or were just qualitative analysis, and majority of these reviews did not include Chinese trials of ESWT for CMPC.

To our best knowledge, this is the first systematic review to explore the effect and safety of ESWT for CMPC. It can provide the updated evidence which is of great importance for patients, clinical practice, and health related policy maker in ESWT treating CMPC.

## Author contributions

**Conceptualization:** Jiawei Qin, Yi Zhang

**Data curation:** Jiawei Qin, Zexiang He, Lijian Wu Formal analysis:

**Investigation:** Zexiang He, Lijian Wu, Qiuxiang Lin, Yiheng Lin

**Methodology:** Jiawei Qin, Tong Jin

**Project administration:** Jiawei Qin

**Resources:** Jiawei Qin, Tong Jin, Yi Zhang

**Software:** Jiawei Qin, Tong Jin

**Supervision:** Jiawei Qin, Tong Jin

**Validation:** Jiawei Qin, Tong Jin, Yi Zhang

**Visualization:** Jiawei Qin, Qiuxiang Lin, Yiheng Lin

**Writing – original draft:** Jiawei Qin, Tong Jin, Yi Zhang, Zexiang He, Lijian Wu

**Writing – review & editing:** Jiawei Qin, Tong Jin, Yi Zhang
